# Treating Recurrent Brain Metastases Using GammaTile Brachytherapy: A Case Report and Dosimetric Modeling Method

**DOI:** 10.7759/cureus.19232

**Published:** 2021-11-03

**Authors:** Theodore Arsenault, Collin M Labak, Kevin Chaung, Raymond F Muzic, Arash Kardan, Andrew Sloan, Serah Choi, Tarun Podder, Zi Ouyang

**Affiliations:** 1 Radiation Oncology, University Hospitals Cleveland Medical Center, Cleveland, USA; 2 Neurosurgery, University Hospitals Cleveland Medical Center, Cleveland, USA; 3 Radiology, University Hospitals Cleveland Medical Center, Cleveland, USA

**Keywords:** dosimetry, brachytherapy, gammatile, reccurent brain metastases, start

## Abstract

One of the treatment options for recurrent brain metastases is surgical resection combined with intracranial brachytherapy. GammaTile® (GT) (GT Medical Technologies, Tempe, Arizona) is a tile-shaped permanent brachytherapy device with cesium 131 (^131^Cs) seeds embedded within a collagen carrier. We report a case of treating a patient with recurrent brain metastases with GT and demonstrate a dosimetric modeling method.

## Introduction

The GammaTile® (GT) (GT Medical Technologies, Tempe, Arizona) is a permanently implantable brachytherapy device which has been introduced for treatment of certain brain tumors [1]. Each GT is 2.0 × 2.0 × 0.4 cm^3^ and holds four cesium 131 (^131^Cs) seeds (Model CS-1 rev2, IsoRay Medcal Inc., Richland, Washington) spaced 1.0 cm apart in a matrix arrangement and embedded within a biocompatible collagen matrix (DuraGen® Suturable Matrix, Integra Lifesciences, Plainsboro, New Jersey). The seeds are 1 mm from the smooth side and 3 mm from the textured side of the tile. The GT is designed for the adherent textured side to be in contact with and conform to the resection cavity. Multiple tiles can be used to cover the surface of the cavity, and each tile can be cut once into a half tile if needed for smaller areas. ^131^Cs in the seeds decays (half-life 9.7 days) by electron capture to xenon 131 (^131^Xe) followed by characteristic x-ray emission with a mean energy 30.411 keV.

Planning and dose evaluation depends on the number and arrangement of GTs. Currently, there is no simple method in GT preplanning or postplan evaluation. Estimation of tile number is guided by the geometric parameters of the tumor, and it is highly dependent on clinical experience [1]. In this study, we propose a simple volume-based dosimetric modeling method, which may provide guidance in GT preplan estimation and postplan evaluation.

## Case presentation

The patient is a 62-year-old right-handed woman with a past medical history of significant tobacco use with chronic obstructive pulmonary disorder, hyperlipidemia, and prior remote meningioma resection at 42 years of age with residual seizures on levetiracetam. She was diagnosed with stage IIB pT1aN1 left lung adenocarcinoma at 51 years of age and underwent left upper lobectomy with mediastinal lymph node dissection followed by adjuvant chemotherapy. Six months after her initial diagnosis, she was found to have brain metastases and underwent four Gamma Knife radiosurgeries to a total of 17 lesions over the course of 12 months. She did well over the next five years but then had progression of a right posterior temporal mass that was in the immediate area of a small lesion previously treated with Gamma Knife. She underwent a right-sided craniotomy and tumor resection, followed by adjuvant stereotactic body radiotherapy (SBRT) with 30 Gy in five fractions to the resection cavity. Two years later, she developed seizures and headaches, and she was found to have a growing focus of enhancement seen using MRI at the site of her previous surgery consistent with disease recurrence. Four months after this finding, she was taken back to the operating room for a redo right craniotomy for tumor resection.

The patient unfortunately once again developed local recurrence at the surgical site seen on fluorodeoxyglucose (FDG) positron emission tomography (PET) and gadolinium-enhanced T1 weighted MRI four months post operation. She was initially considered for laser interstitial thermal therapy (LITT) but was thought to have a tumor configuration and morphology that is not amenable for treatment using this modality. After multi-disciplinary tumor board discussion, she was ultimately recommended repeat resection and GT. Accordingly, the patient underwent a third right craniotomy with tumor resection and placement of GTs in the tumor bed, and titanium mesh cranioplasty overlying the surgical site. The patient tolerated the procedure well.

A target was contoured on the preoperative MRI by the radiation oncologist. The prescription was to treat 60 Gy to 5 mm beyond the surgical cavity. The number of tiles needed was estimated by evaluating the circumference of the target in the axial, coronal, and sagittal views. Five tiles were estimated by a team consisting of the neurosurgeon, radiation oncologist, and medical physicist for the procedure. An extra sixth tile was ordered to account for any changes in the cavity on the day of the surgery. Three full and three half tiles were implanted, resulting in a total of 18 seeds with 3.51 U (5.51 mCi) per seed.

**Figure 1:**
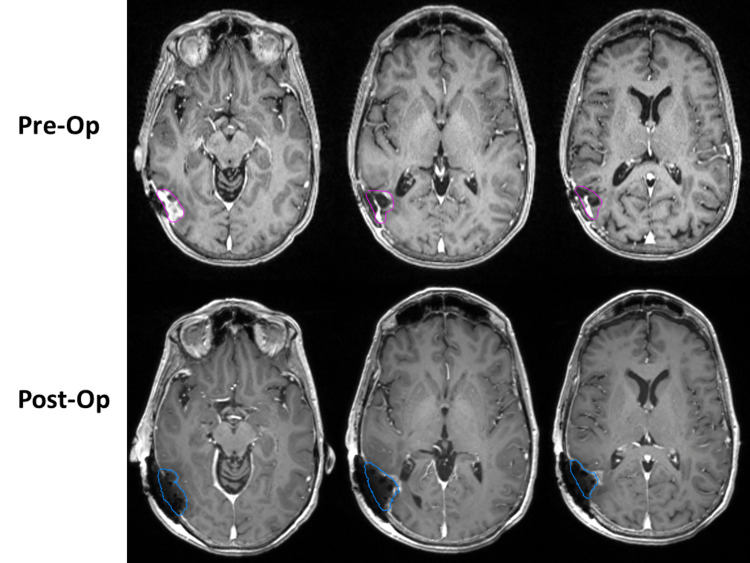
Gadolinium-enhanced volumetric MRIs with the target and surgical cavity contours. The top row shows the preoperative (pre-op) images and the bottom row shows the postoperative (post-op) images.

The patient had a thin slice (1 mm) CT of the head on postoperative day zero, which was unremarkable (i.e., no acute hemorrhage); MRI on postoperative day two showed a near-total resection. She also had a two-week follow-up wound check and was doing well at that time. The CT and MRI were fused, the postoperative cavity was contoured, and the ^131^Cs seeds were identified. A 5-mm expansion from the cavity (excluding bone) was used as a clinical target volume (CTV). The postoperative cavity volume was 14.36 cm^3^. Figure 1 shows preoperative and postoperative MRI with cavity contours.

**Figure 2:**
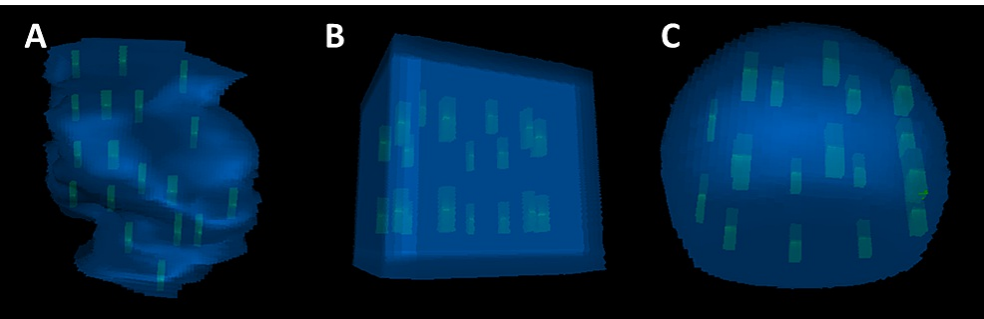
Three-dimensional representation of (A) real patient cavity, (B) cube cavity, and (C) spherical cap cavity with 18 GT seeds in each.

To model the GT dosimetry on a phantom CT, we created a spherical cap and a cube with the same volume as the postoperative cavity, expanded 5 mm (in all directions except for one flat surface), and acquired the CTV. Four and a half tiles (18 seeds in total) were each modeled in the spherical cap and the cube according to the tile configuration. The seeds were 3 mm from the cavity wall and 1 cm from each other. Figure 2 shows the three-dimensional representation of the 18 seeds in the postoperative cavity for the patient and for the cube and spherical cap models.

**Figure 3:**
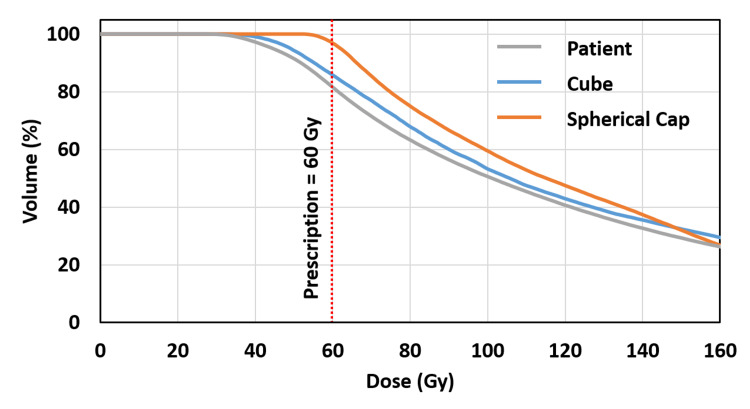
DVHs for the CTV in postplans for the patient (gray), cube model (blue), and spherical cap model (orange). The red vertical line represents the prescription dose at 60 Gy. The cube model resembles the patient DVH more closely than the spherical cap model. DVH: dose volume histogram; CTV: clinical target volume

Postoperative plans for the patient and the two models were planned in MIM Symphony™ (MIM Software Inc., Cleveland, Ohio). Dose volume histograms (DVH) were compared for all three plans (Figure 3). The cube model resembled the patient DVH better than the spherical cap model. Table 1 shows the dosimetry parameters of the patient and the two models. Volume of the contours, mean dose, CTV volume receiving the prescription dose (V_60Gy_), and dose to 90% of the CTV (D_90%_) are reported.

**Table 1:** Dosimetric parameters of the patient and simplified models. *Cavity volume should ideally be the same for the patient and the two models. The slight differences are due to finite voxel resolution and rounding.

	Contour volume		CTV dosimetry		
	Cavity (cm^3^)*	CTV (cm^3^)	Mean Dose (Gy)	V_60Gy_ (%)	D_90%_ (Gy)
Patient	14.36	29.82	131.02	81.55	51.80
Cube	14.12	31.95	136.55	85.77	55.20
Spherical Cap	14.48	30.48	136.00	96.86	66.20

## Discussion

In this case study, the cube model outperformed the spherical cap model as shown in Table 1 and Figure 3. The cube model overestimates the mean dose by 5.53 Gy, V_60Gy_ by 4.22%, and D_90%_ by 3.4 Gy; the spherical cap model overestimates the mean dose by 4.98 Gy, V_60Gy_ by 15.31%, and D_90%_ by 14.4 Gy. The spherical cap represents an ideal scenario, where the cavity shape is smooth and there is no sharp edges or hard-to-reach corners. In reality, this is rarely true. As shown in Figure 2, the patient cavity has an irregular shape and many edges and corners, which is why the patient DVH shows a lower dose coverage than the spherical cap model. On the other hand, the cube model represents a less ideal and yet simple scenario. The edges and corners of the cube contribute to the under-coverage in the DVH, and the simple shape of the cube allows for quick dosimetry analysis.

Several studies reported treating patients with brain tumors with GT [1-3], including gliomas, metastases, meningiomas, and glioblastomas. It is noted that dosimetric analysis of GT is limited in published literature. Ferreira et al. reported that no nomogram or lookup table is available for GT dosimetry, and the tile number can be estimated based on the surface area of the surgical bed [1]. As observed in our case, simple volume-based dosimetric models may exist, which may provide guidance in GT preplan, tile estimation, and postplan quality assurance.

## Conclusions

Treatment planning for GT is not fully studied or standardized. We demonstrate a simple dosimetric modeling method with the cube model showing good agreement with the patient dosimetry, which warrants an extended study with a larger number of patients in the future. This method may provide further guidance to institutions implementing GT therapy.

## Figures and Tables

**Figure 4:**
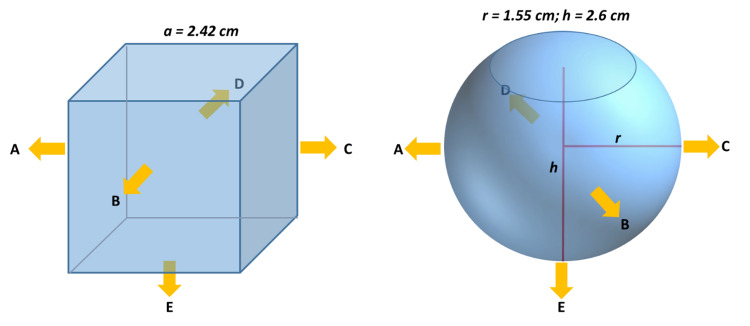
Specifications of the cube and spherical cap models. The yellow arrows indicate the directions of clinical target volume (CTV) expansions. A, B, C, D, and E are the tiled surfaces.

## Appendices

Specifications of the cube and spherical cap models

Figure 4 shows the details of the cube and spherical cap models used in this case study. The cube has a side length *a *= 2.42 cm. The spherical cap has a radius *r *= 1.55 cm, and a height *h *= 2.6 cm. To obtain the CTVs, as shown by the yellow arrows, the cavity models are expanded in all directions by 5 mm except for the top flat surface, which represents the cavity opening. A, B, C, D, and E represent the five tiled surfaces. A, B, C, and D each have one tile, and E has a half tile.

A theoretical example of using the cube model to estimate tiles

In this case study, the radiation oncologist contoured a pre-op cavity based on the pre-op MRI (Figure 1). The pre-op cavity volume was 8.08 cm^3^, which corresponded to a cube with side length *a *= 2 cm. For 2 cm ≤ a < 3 cm, one tile is assigned to each of the five tiled surfaces, resulting in an estimation of five tiles.
